# Crystal structure of di-μ-iodido-bis­[bis(aceto­nitrile-κ*N*)copper(I)]

**DOI:** 10.1107/S2056989015018149

**Published:** 2015-10-03

**Authors:** Eva Rebecca Barth, Christopher Golz, Michael Knorr, Carsten Strohmann

**Affiliations:** a Fakultät für Chemie und Chemische Biologie, Technische Universität Dortmund, Otto-Hahn-Strasse 6, 44227 Dortmund, Germany; b Institut UTINAM, UMR CNRS 6213, Université de Franche-Comté, 16 Route de Gray, 25030 Besançon, France

**Keywords:** crystal structure, copper(I) iodide complex, dimer

## Abstract

The title compound, [Cu_2_I_2_(CH_3_CN)_4_], exhibits a centrosymmetric Cu_2_I_2_ core [Cu⋯Cu distance = 2.7482 (11) Å], the Cu^I^ atoms of which are further coordinated by four mol­ecules of aceto­nitrile. The Cu^I^ atom has an overall distorted tetra­hedral coordination environment evidenced by *L*—Cu—*L* angles (*L* = N or I) ranging from 100.47 (10) to 117.06 (2)°. The coordination geometries of the aceto­nitrile ligands deviate slightly from linearity as shown by Cu—N—C angles of 167.0 (2) and 172.7 (2)°. In the crystal, there are no significant hydrogen-bonding inter­actions present, so the crystal packing seems to be formed predominantly by van der Waals forces.

## Related literature   

The title mol­ecule is the active species used for the synthesis of luminescent compounds with (CuI)_*n*_ moieties, prepared by treatment with sulfur ligands. For more details of syntheses and properties of these compounds, see: Knorr *et al.* (2012 ▸, 2014 ▸, 2015 ▸). The N—Cu—N angle of 100.47 (10)° in the title compound is comparable with that found in the pyridine-coordinated CuI dimer (Dyason *et al.*, 1984 ▸). For crystal structures of CuI dimers with additional di­amine ligands, see: Haitko (2007 ▸); Garbauskas *et al.* (1986 ▸). The title compound represents, to the best of our knowledge, the first crystallographic characterization of CuI with aceto­nitrile as the only co-ligand, probably caused by the sensitive nature of the crystals. In the solid state, CuI appears as a polymer (Wyckoff & Posnjak, 1922 ▸).
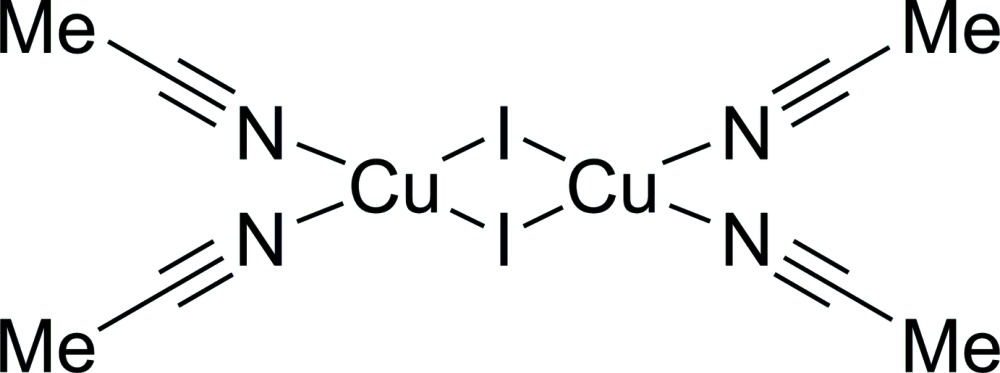


## Experimental   

### Crystal data  

[Cu_2_I_2_(C_2_H_3_N)_4_]
*M* *_r_* = 545.10
Monoclinic 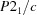

*a* = 7.669 (3) Å
*b* = 14.367 (5) Å
*c* = 7.944 (3) Å
β = 116.957 (5)°
*V* = 780.2 (5) Å^3^
*Z* = 2
Mo *K*α radiation
μ = 6.66 mm^−1^
*T* = 173 K
0.4 × 0.3 × 0.2 mm

### Data collection  

Bruker APEXII CCD diffractometer
Absorption correction: multi-scan (*SADABS*; Bruker, 2003 ▸) *T*_min_ = 0.178, *T*_max_ = 1
18211 measured reflections
1696 independent reflections
1628 reflections with *I* > 2σ(*I*)
*R*_int_ = 0.052

### Refinement  

*R*[*F* ^2^ > 2σ(*F* ^2^)] = 0.021
*wR*(*F* ^2^) = 0.056
*S* = 1.16
1696 reflections
76 parameters
H-atom parameters constrained
Δρ_max_ = 0.77 e Å^−3^
Δρ_min_ = −0.70 e Å^−3^

### 

Data collection: *APEX2* (Bruker, 2003 ▸); cell refinement: *SAINT* (Bruker, 2003 ▸); data reduction: *SAINT* ; program(s) used to solve structure: *SHELXS2014* (Sheldrick, 2008 ▸); program(s) used to refine structure: *SHELXL2014* (Sheldrick, 2015 ▸); molecular graphics: *SHELXTL* (Sheldrick, 2008 ▸); software used to prepare material for publication: *OLEX2* (Dolomanov *et al.*, 2009 ▸) and *publCIF* (Westrip, 2010 ▸).

## Supplementary Material

Crystal structure: contains datablock(s) I. DOI: 10.1107/S2056989015018149/wm5208sup1.cif

Structure factors: contains datablock(s) I. DOI: 10.1107/S2056989015018149/wm5208Isup2.hkl

**Fig. 1.**
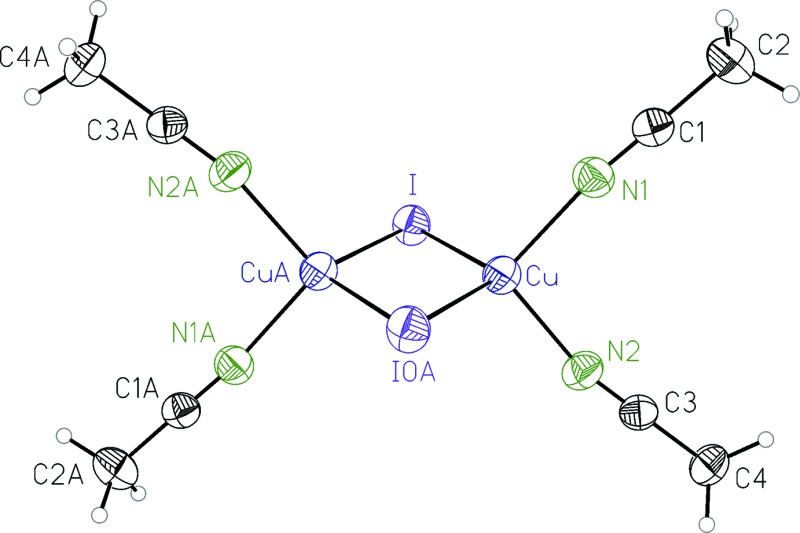
The molecular structure of the title compound with anisotropic displacement ellipsoids drawn at the 50% probability level.

. DOI: 10.1107/S2056989015018149/wm5208fig1.tif
The mol­ecular structure of the title compound with anisotropic displacement ellipsoids drawn at the 50% probability level.

**Fig. 2.**
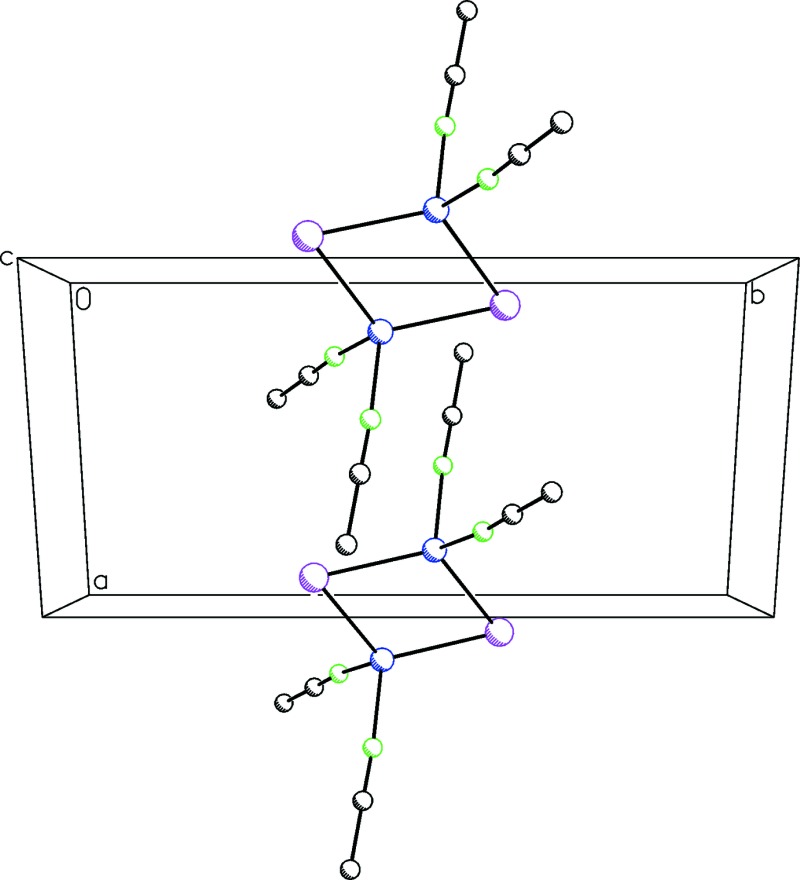
Part of the crystal packing of the title compound viewed along [001]. H atoms were omitted for clarity.

. DOI: 10.1107/S2056989015018149/wm5208fig2.tif
Part of the crystal packing of the title compound viewed along [001]. H atoms were omitted for clarity.

CCDC reference: 1427944

Additional supporting information:  crystallographic information; 3D view; checkCIF report

## supplementary crystallographic information

### S1. Synthesis and crystallization

The title compound occured as a byproduct from the synthesis of copper(I) iodide compounds (see: *Related literature*). CuI was dissolved in aceto­nitrile and treated with an excess of sulfur ligands (*i.e.* di­methyl sulfide). After stirring, the solution was stored in a refridgerator over night, followed by keeping it in a freezer (Knorr *et al.*, 2015). In addition to the crystallized CuI compounds, the title compound could be isolated in traces.

### S2. Refinement

Methyl hydrogen atoms were set geometrically and refined as a rotating group with *U*_iso_(H) = 1.5*U*_eq_(C).

### Crystal data

**Table:**

[Cu_2_I_2_(C_2_H_3_N)_4_]	*F*(000) = 504
*M**_r_* = 545.10	*D*_x_ = 2.320 Mg m^−^^3^
Monoclinic, *P*2_1_/*c*	Mo *K*α radiation, λ = 0.71073 Å
*a* = 7.669 (3) Å	Cell parameters from 5543 reflections
*b* = 14.367 (5) Å	θ = 2.8–27.1°
*c* = 7.944 (3) Å	µ = 6.66 mm^−^^1^
β = 116.957 (5)°	*T* = 173 K
*V* = 780.2 (5) Å^3^	Block, colourless
*Z* = 2	0.4 × 0.3 × 0.2 mm

### Data collection

**Table:**

Bruker APEXII CCD diffractometer	1628 reflections with *I* > 2σ(*I*)
Graphite monochromator	*R*_int_ = 0.052
phi and ω scans	θ_max_ = 27.0°, θ_min_ = 2.8°
Absorption correction: multi-scan (*SADABS*; Bruker, 2003)	*h* = −9→9
*T*_min_ = 0.178, *T*_max_ = 1	*k* = −18→18
18211 measured reflections	*l* = −10→10
1696 independent reflections	

### Refinement

**Table:**

Refinement on *F*^2^	Hydrogen site location: inferred from neighbouring sites
Least-squares matrix: full	H-atom parameters constrained
*R*[*F*^2^ > 2σ(*F*^2^)] = 0.021	*w* = 1/[σ^2^(*F*_o_^2^) + (0.0281*P*)^2^ + 0.2851*P*] where *P* = (*F*_o_^2^ + 2*F*_c_^2^)/3
*wR*(*F*^2^) = 0.056	(Δ/σ)_max_ < 0.001
*S* = 1.16	Δρ_max_ = 0.77 e Å^−^^3^
1696 reflections	Δρ_min_ = −0.70 e Å^−^^3^
76 parameters	Extinction correction: *SHELXL2014* (Sheldrick, 2015), Fc^*^=kFc[1+0.001xFc^2^λ^3^/sin(2θ)]^-1/4^
0 restraints	Extinction coefficient: 0.0356 (10)

### Special details

**Table:**

Geometry. All e.s.d.'s (except the e.s.d. in the dihedral angle between two l.s. planes) are estimated using the full covariance matrix. The cell e.s.d.'s are taken into account individually in the estimation of e.s.d.'s in distances, angles and torsion angles; correlations between e.s.d.'s in cell parameters are only used when they are defined by crystal symmetry. An approximate (isotropic) treatment of cell e.s.d.'s is used for estimating e.s.d.'s involving l.s. planes.
Refinement. 1. Fixed *U*_iso_ At 1.5 times of: All C(H,H,H) groups 2.a Idealized Me refined as rotating group: C2(H2A,H2B,H2C), C4(H4A,H4B,H4C)

### Fractional atomic coordinates and isotropic or equivalent isotropic displacement parameters (Å^2^)

**Table:**

	*x*	*y*	*z*	*U*_iso_*/*U*_eq_	
C1	0.2924 (4)	0.35511 (17)	0.2436 (4)	0.0327 (5)	
C2	0.3602 (5)	0.3046 (2)	0.1258 (5)	0.0477 (7)	
H2A	0.3147	0.2400	0.1117	0.072*	
H2B	0.5035	0.3057	0.1851	0.072*	
H2C	0.3081	0.3341	0.0012	0.072*	
C3	0.5869 (4)	0.43439 (18)	0.8689 (4)	0.0327 (5)	
C4	0.7870 (4)	0.4169 (2)	1.0081 (4)	0.0440 (6)	
H4A	0.7881	0.3711	1.1000	0.066*	
H4B	0.8464	0.4750	1.0734	0.066*	
H4C	0.8617	0.3927	0.9451	0.066*	
Cu	0.17322 (5)	0.46160 (2)	0.52217 (4)	0.03798 (12)	
I	−0.09223 (2)	0.36402 (2)	0.57402 (3)	0.03945 (11)	
N1	0.2374 (3)	0.39446 (16)	0.3335 (3)	0.0386 (5)	
N2	0.4306 (3)	0.44856 (16)	0.7589 (3)	0.0402 (5)	

### Atomic displacement parameters (Å^2^)

**Table:**

	*U*^11^	*U*^22^	*U*^33^	*U*^12^	*U*^13^	*U*^23^
C1	0.0351 (13)	0.0281 (12)	0.0329 (13)	−0.0024 (9)	0.0137 (11)	0.0016 (9)
C2	0.0589 (18)	0.0430 (15)	0.0494 (17)	−0.0014 (13)	0.0318 (15)	−0.0063 (13)
C3	0.0348 (13)	0.0328 (12)	0.0294 (11)	−0.0015 (10)	0.0137 (10)	0.0003 (10)
C4	0.0323 (13)	0.0523 (17)	0.0381 (14)	0.0054 (12)	0.0077 (11)	0.0013 (12)
Cu	0.03422 (19)	0.0400 (2)	0.03415 (19)	0.00505 (13)	0.01066 (14)	−0.00146 (13)
I	0.03990 (14)	0.03205 (13)	0.04649 (15)	0.00095 (6)	0.01965 (10)	0.00337 (6)
N1	0.0407 (12)	0.0370 (12)	0.0369 (12)	0.0027 (10)	0.0164 (10)	−0.0009 (10)
N2	0.0345 (12)	0.0438 (13)	0.0353 (11)	0.0002 (10)	0.0096 (10)	0.0007 (10)

### Geometric parameters (Å, º)

**Table:**

C1—C2	1.454 (4)	C4—H4B	0.9800
C1—N1	1.132 (4)	C4—H4C	0.9800
C2—H2A	0.9800	Cu—Cu^i^	2.7482 (11)
C2—H2B	0.9800	Cu—I^i^	2.6108 (10)
C2—H2C	0.9800	Cu—I	2.6532 (8)
C3—C4	1.450 (3)	Cu—N1	2.022 (2)
C3—N2	1.137 (3)	Cu—N2	2.025 (2)
C4—H4A	0.9800	I—Cu^i^	2.6108 (10)
			
N1—C1—C2	179.2 (3)	I—Cu—Cu^i^	57.78 (3)
C1—C2—H2A	109.5	I^i^—Cu—Cu^i^	59.286 (14)
C1—C2—H2B	109.5	I^i^—Cu—I	117.06 (2)
C1—C2—H2C	109.5	N1—Cu—Cu^i^	129.99 (7)
H2A—C2—H2B	109.5	N1—Cu—I	108.95 (7)
H2A—C2—H2C	109.5	N1—Cu—I^i^	110.27 (7)
H2B—C2—H2C	109.5	N1—Cu—N2	100.47 (10)
N2—C3—C4	179.4 (3)	N2—Cu—Cu^i^	129.41 (8)
C3—C4—H4A	109.5	N2—Cu—I	107.48 (8)
C3—C4—H4B	109.5	N2—Cu—I^i^	111.28 (7)
C3—C4—H4C	109.5	Cu^i^—I—Cu	62.94 (2)
H4A—C4—H4B	109.5	C1—N1—Cu	172.7 (2)
H4A—C4—H4C	109.5	C3—N2—Cu	167.0 (2)
H4B—C4—H4C	109.5		

Symmetry code: (i) −*x*, −*y*+1, −*z*+1.
