# Supplementary figures and images for: Crystal structure of di-μ-iodido-bis­[bis(aceto­nitrile-κN)copper(I)]
Source: Acta Crystallogr E Crystallogr Commun. 2015 Oct 3;71(Pt 11):m189–90. doi: 10.1107/S2056989015018149 (PMC4645014; doi:10.1107/S2056989015018149)

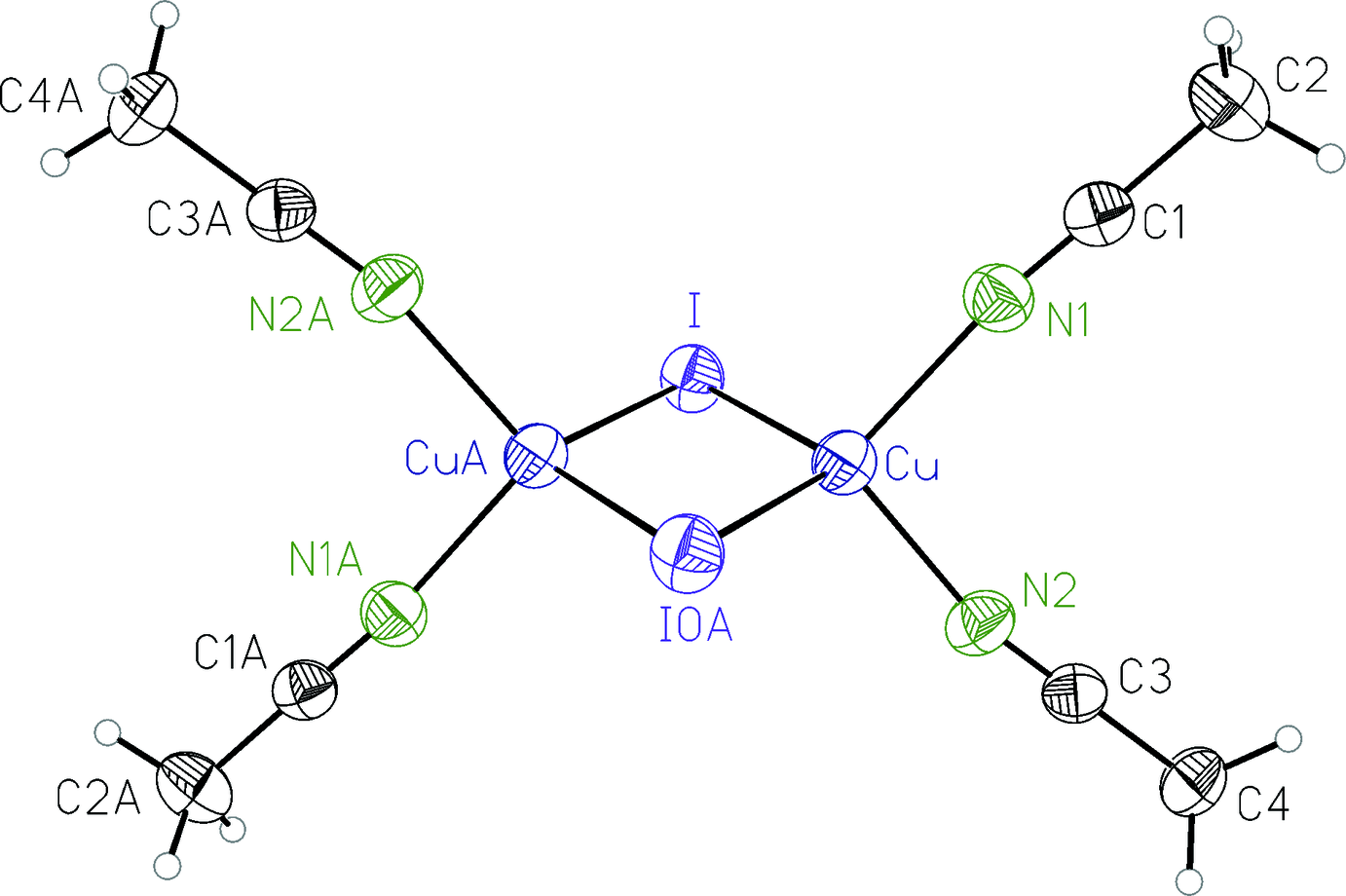

Supplement: Supplementary file 3 [file e-71-0m189-fig1.tif]

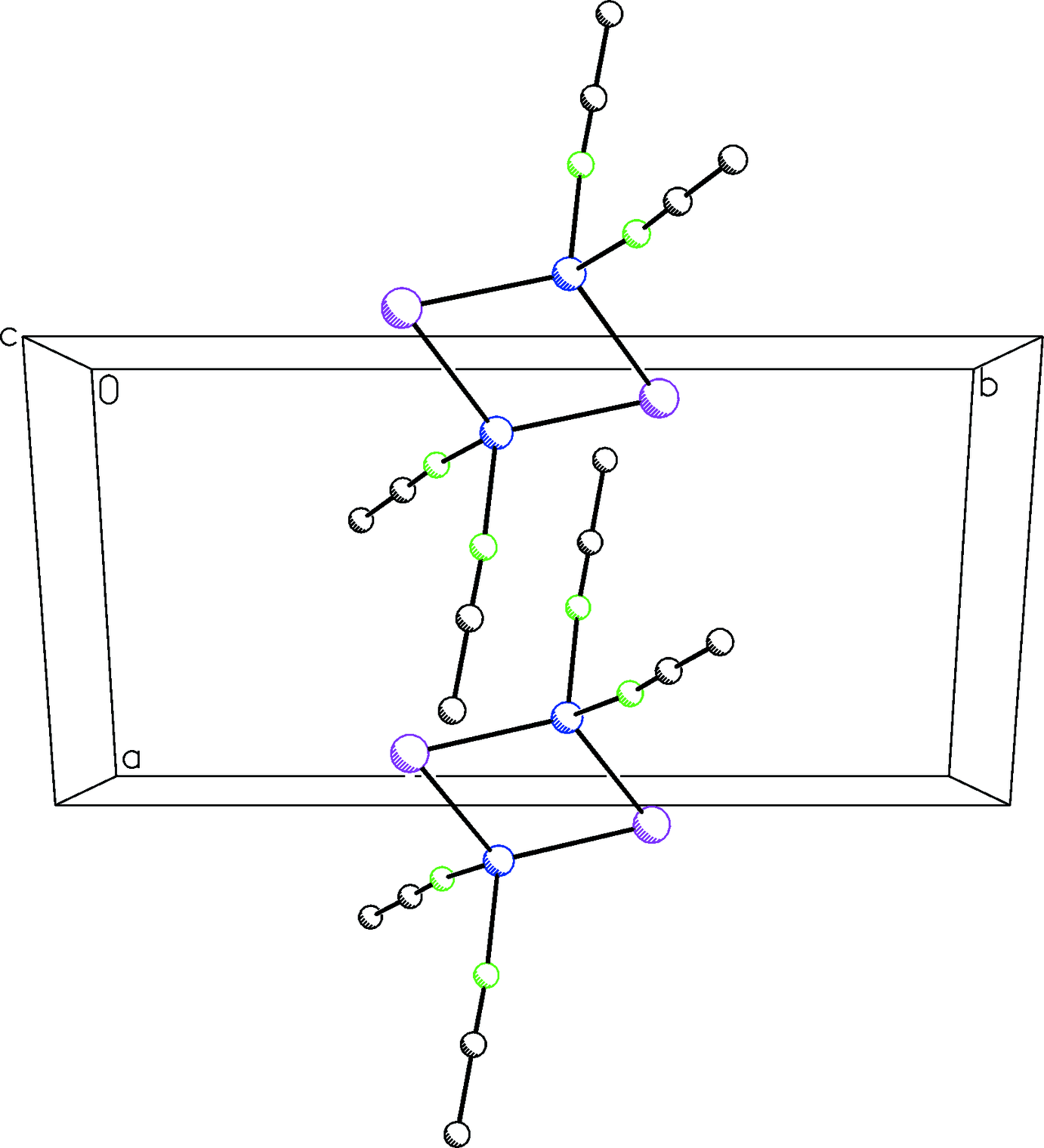

Supplement: Supplementary file 4 [file e-71-0m189-fig2.tif]
